# Multiomics landscape of the autosomal dominant osteopetrosis type II disease-specific induced pluripotent stem cells

**DOI:** 10.1186/s41065-021-00204-x

**Published:** 2021-10-27

**Authors:** Chunhong Li, Yu Shangguan, Peng Zhu, Weier Dai, Donge Tang, Minglin Ou, Yong Dai

**Affiliations:** 1 Central Laboratory, Guangxi Health Commission Key Laboratory of Glucose and Lipid Metabolism Disorders, The Second Affiliated Hospital of Guilin Medical University, Guilin, Guangxi 541199 People’s Republic of China; 2 Clinical Medical Research Center, Guangdong Provincial Engineering Research Center of Autoimmune Disease Precision Medicine, Shenzhen Engineering Research Center of Autoimmune Disease, The Second Clinical Medical College of Jinan University, The First Affiliated Hospital of Southern University of Science and Technology, Shenzhen People’s Hospital, Shenzhen, Guangdong 518020 People’s Republic of China; 3 Guangxi Key Laboratory of Metabolic Disease Research, Central Laboratory of Guilin, NO. 924 Hospital, Guilin, 541002 People’s Republic of China; 4 College of Natural Science, University of Texas at Austin, Austin, TX 78712 USA

**Keywords:** Osteopetrosis, Osteoclast, Whole genome sequencing, DNA methylation, N6-methyladenosine

## Abstract

**Background:**

Autosomal dominant osteopetrosis type II (ADO2) is a genetically and phenotypically metabolic bone disease, caused by osteoclast abnormalities. The pathways dysregulated in ADO2 could lead to the defects in osteoclast formation and function. However, the mechanism remains elusive.

**Materials and methods:**

To systematically explore the molecular characterization of ADO2, we performed a multi-omics profiling from the autosomal dominant osteopetrosis type II iPSCs (ADO2-iPSCs) and healthy normal control iPSCs (NC-iPSCs) using whole genome re-sequencing, DNA methylation and N6-methyladenosine (m6A) analysis in this study.

**Results:**

Totally, we detected 7,095,817 single nucleotide polymorphisms (SNPs) and 1,179,573 insertion and deletions (InDels), 1,001,943 differentially methylated regions (DMRs) and 2984 differential m6A peaks, and the comprehensive multi-omics profile was generated from the two cells. Interestingly, the ISG15 m6A level in ADO2-iPSCs is higher than NC-iPSCs by IGV software, and the differentially expressed m6A-modified genes (DEMGs) were highly enriched in the osteoclast differentiation and p53 signaling pathway, which associated with the development of osteopetrosis. In addition, combining our previously published transcriptome and proteome datasets, we found that the change in DNA methylation levels correlates inversely with some gene expression levels.

**Conclusion:**

Our results indicate that the global multi-omics landscape not only provides a high-quality data resource but also reveals a dynamic pattern of gene expression, and found that the pathogenesis of ADO2 may begin early in life.

**Supplementary Information:**

The online version contains supplementary material available at 10.1186/s41065-021-00204-x.

## Introduction

Autosomal dominant osteopetrosis type II (ADO2) is a rare human inherited metabolic bone disease characterized by increased bone brittleness, mass and density due to osteoclast abnormalities [1–3]. It has a prevalence estimated at 1 in 20,000 live births, and usually occurs in late childhood or adolescence, but can also be detected in early infancy [2]. However, there is no curative therapeutics for ADO2 [4]. In most patients, they result from heterozygous mutations in CLCN7, which can result in nerve compression syndrome, visual loss and bone marrow failure, and may be presented diverse clinical and radiological manifestations ranging from asymptomatic or relatively mild symptoms to the very severe phenotype [2, 5]. Notably, some promising results suggest that small interfering RNA (siRNA)-based therapeutics may be used to cure patients with ADO2 and have been further demonstrated to be feasible in osteoporotic animal models [6, 7].

Currently, the integrative analysis of multi-omics has provided new ways for biomedical research, which can explain many complicated biological events associated with diseases [8–11]. Some evidence has strongly indicated that molecular changes at the DNA, RNA and protein levels could change the microenvironment of osteoclastogenesis, and lead to the defects in osteoclast formation and function [1, 12, 13]. To date, large-scale whole exome-sequenced studies of osteopetrosis have revealed more than 50 heterozygous mutations in the CLCN7 gene, which can lead to ADO2, among which the p.G215R has been studied extensively [13–15]. Ou and his colleagues have been identified a great number of miRNAs and proteins in the peripheral blood mononuclear cells from patients with osteopetrosis, and found that the changes in miRNA expression profiles suggest epigenetic variation [12]. A recent transcriptomic study has been suggested that changes in the expression of ITGB5, PRF1, WARS, and SERPINE2 may be part of the osteoclast phenotype, in addition to the acidification dysfunction caused by heterozygous mutations in the CLCN7 gene in osteoclasts in ADO2 patients [13]. In the past, knowledge about the molecular characterization and pathogenesis of osteopetrosis was primarily acquired by osteoporotic animal models [5, 16–19]. Recently, osteoporotic iPSC models were successfully generated from an ADO2 patient and three autosomal recessive osteopetrosis (ARO) patients, which has provided an unprecedented opportunity for the study of ADO2 pathophysiology and therapeutic application [1, 20, 21]. And thus, the molecular characterization of ADO2 is urgently to discover underlying biomarkers for osteopetrosis, facilitate early diagnosis, and accelerate the development of personalized therapeutics.

Here, we used the multi-omics combined with bioinformatics analysis to establish a multi-omics landscape, and observed a widespread epigenetic and transcription-regulatory pattern in the ADO2-iPSCs. In addition, our results indicate that the global multi-omics landscape not only provides a high-quality data resource but also reveals a dynamic pattern of gene expression, and found that the pathogenesis of ADO2 may begin early in life.

## Materials and methods

### Cell samples

Written informed consent was obtained from the 31-year-old male participant who was diagnosed with ADO2 and was further confirmed to carry a mutation in CLCN7 (R286W). The urine cells of this participant were reprogrammed to generate ADO2-iPSC colonies, and the best colony was selected to construct the ADO2-iPS cell line [1]. After the morphology of the cell line was identified, cells were collected, immediately transferred into liquid nitrogen and stored for future use. The healthy normal control iPSCs (NC-iPSCs) were provided by Cellapy Biotechnology (Beijing, China). This study was reviewed and approved by the Ethics Committee of Shenzhen People’s Hospital (LL-KT-201801127).

### Whole genome library construction and sequencing

Total DNA was extracted from autosomal dominant osteopetrosis type II iPSCs (ADO2-iPSCs) and normal control iPSCs (NC-iPSCs) using TIANamp Genomic DNA Kit (Cat. #DP304–02, Lot#03427, TIANGEN BIOTECH (BEIJING) CO., LTD) according to the manufacturer’s protocol. Agarose gel electrophoresis was used to analyze the degree of DNA degradation and the presence of stray bands, RNA and protein contamination. The quantity of total DNA was measured by a NanoDrop 2000 spectrophotometer (Thermo Fisher Scientific, Inc., Waltham, MA, USA) and Qubit® 2.0 Fluorometer (Thermo Fisher Scientific, Inc.), following which the total DNA samples with A260/280 ratios located range 1.8 to 2.0 were used for whole genome and DNA methylation library construction. The total DNA samples were randomly fragmented by a Covaris S220 Focused UItrasonicator (Covaris, Woburn, MA, USA) into 350 bp fragments. The sheared products were detected by an Agilent 2100 Bioanalyzer system (Agilent, G2939AA). After the ends of the DNA fragments were repaired and an Illumina Adaptor was added, the PCR was used for whole genome library construction. And then, the qualified libraries were sequenced by an Illumina Novaseq™ 6000 sequencer at LC-BIO (Hangzhou, China) with 150 base paired-end reads.

### SNPs and InDels detection and annotation

The advanced bioinformatics analysis began with raw data generated from the NovaSeq6000 platforms. The sequence signatures with adapter ligation, low quality, and containing > 5 unknown bases were removed to obtain clean sequences, which were subjected to further analysis. The clean sequences were aligned to the human reference genome (GRCh38) by a Burrows-Wheeler Aligner (Version 0.7.8-r455) [22], and the alignment results were saved in BAM format files. For further advanced analysis of the final BAM files, single nucleotide polymorphisms (SNPs) and insertion and deletions (InDels) were detected by SAMtools (Version 1.0) [23]. Sambamba (Version 0.6.6) [24] was used to mark duplicate reads. Finally, ANNOVAR (Version 2017Jul16) [25] was used for annotation and classification.

### DNA methylated library construction and sequencing

After total DNA extraction in the previous step, the DNA samples were randomly fragmented by a Covaris S220 Focused UItrasonicator (Covaris, Woburn, MA, USA) for end-repair and adapter ligation. The ends of the sheared DNA fragments were repaired and an Illumina Adaptor was added by using Accel-NGS Methyl-Seq DNA Library Kit (Swift, MI, USA), and then they were subjected to bisulfite conversion. After the PCR was performed, the products were purified using Bead-based SPRI and sequenced on an Illumina Hiseq 4000 sequencer at LC-BIO (Hangzhou, China) with 150 base paired-end reads.

### DNA methylation differential analysis

The raw sequencing datasets were initially processed by a perl scripts in house and Cutadapt [26], to remove the reads containing sequencing adaptors, low quality bases and undetermined bases. Then, the quality of expressed sequence tags was conducted using FastQC, and the valid reads were mapped to the human reference genome (GRCh38) by WALT [27]. And then, The DNA methylation levels were measured by the ratio of the number of reads supporting C (methylated) to that of total reads (methylated and unmethylated). The differential methylated regions (DMRs) between the two cell lines were detected using R package-MethylKit according to *p* value < 0.05, and were subjected to performed volcano plot filtering, GO and KEGG enrichment pathway analyses.

### RNA methylated library construction and sequencing

Total RNA was extracted from the two cell lines using TRIzol reagent (Invitrogen, CA, USA) according to the manufacturer’s protocol. After total RNA extraction, four RNA methylated libraries were constructed as previously reported, with minor modifications [28]. Briefly, the quantity and purity of RNA samples were analyzed by a Bioanalyzer 2100 and RNA 6000 Nano LabChip Kit (Agilent, CA, USA) with RIN number > 7.0. Then, the Poly (A) mRNA was isolated from approximately more than 200 μg of total RNA using poly-T oligo attached magnetic beads (Invitrogen). Following purification, the poly(A) mRNA fractions is fragmented into ~ 100-nt-long oligonucleotides using divalent cations under elevated temperature. Then the sheared RNA fragments were subjected to incubated for 2 h at 4 °C with m6A-specific antibody (No. 202003, Synaptic Systems, Germany) in IP buffer (50 mM Tris-HCl, 750 mM NaCl and 0.5% Igepal CA-630) supplemented with BSA (0.5 μg μl − 1). The mixture was then incubated with protein-A beads and eluted with elution buffer (1 × IP buffer and 6.7 mM m6A). Eluted RNA was precipitated by 75% ethanol. Eluted m6A-containing fragments (IP) and untreated input control fragments are converted to final cDNA library in accordance with a strand-specific library preparation by dUTP method. The average insert size for the paired-end libraries was ~ 100 ± 50 bp. And then, the qualified libraries were sequenced by an Illumina Novaseq™ 6000 sequencer at LC-BIO (Hangzhou, China) with 150 base paired-end reads.

### RNA methylation differential analysis

The raw sequencing datasets were initially processed by a perl scripts in house and Cutadapt [26], to remove the reads containing sequencing adaptors, low quality bases and undetermined bases. Then, the quality of expressed sequence tags was conducted using FastQC, and the valid reads were mapped to the human reference genome (GRCh38) by HISAT2 [29]. Mapped reads of IP and input libraries were provided for R package exomePeak [30], which identifies m6A peaks with bed or bam format that can be adapted for visualization on the UCSC genome browser or IGV software (http://www.igv.org/). MEME [31] and HOMER [32] were used for de novo and known motif finding followed by localization of the motif with respect to peak summit by perl scripts in house. Called peaks were annotated by intersection with gene architecture using ChIPseeker [33]. Then StringTie [34] was used to perform expression level for all mRNAs from input libraries by calculating FPKM. The differentially expressed mRNAs were selected with log2 (fold change) > 1 or log2 (fold change) < − 1 and *p* value < 0.05 by R package edgeR [35].

### qRT-PCR analysis

Quantitative real-time polymerase chain reaction (qRT-PCR) was used to verify the identified mRNAs and miRNAs from the sequencing results in the ADO2-iPSCs and NC-iPSCs. Total RNA was extracted from the two cell lines described above and subjected to first-strand cDNA synthesis using the TRUEscript 1st Stand cDNA SYNTHESIS Kit (Aidlab, Beijing, China). The specific primers used in the qRT-PCR are listed in Table 1, and qRT-PCR was performed using a SYBR PrimeScript miRNA RT-PCR Kit (TianGen Biotech, Beijing, China). Each qRT-PCR mix was as follows: 5 μl 2 × SYBR® Green Supermix, 0.5 μl specific forward primer, 0.5 μl specific reverse primer, 1 μl cDNA template and 3 μl ddH2O. The qRT-PCR procedure was as follows: 95 °C for 3 min followed by 39 cycles of 95 °C for 10 s and 60 °C for 30 s, with a melt curve analysis. GAPDH and U6 was used as an internal control gene for mRNAs and miRNAs, respectively. Three independent biological replicates were performed for each gene. The qRT-PCR data were analyzed by a comparative 2-ΔΔCt method [36], and the average of three independent experiments for each gene was calculated as the relative expression level by GraphPad Prism (version 8.0.1).

**Table 1:** The primers using for PCR amplification

NO.	Target	Sequence	TM (°C)
1	ENST00000162749	F: CTGTACGCCGTGGTGGAGAAC R: TCGTGGTCGCTCAGCCCTA	60
2	ENST00000398795	F: ATCAGAGCAGAGAAAGCGATGG R: GATGGGATGTCGGTGGCATTA	60
3	ENST00000329608	F: TAGGCAACGAGCGACAGG R: GTAGGGCAGGGTCTCTTAGC	60
4	ENST00000316804	F: CTCCGACTCCAGATATGAACAG R: GAGAAGCAGGATGAGGAAGAG	60
5	ENST00000285379	F: GCCTCCTTCCTGAATCCTTGG R: CTGATGGGTTCCTTGAGCACAA	60
6	ENST00000382745	F: AGAAGGTCGGCGTCATTGTG R: GGCAGGCTGGGTGTCATC	60
7	ENST00000193322	F: GGTGGTAAGTGCCTGTAGTCCTA R: GCAGTGGTACAATCTTGGCTCAT	60
8	hsa-miR-29a-3p	F: CGGCGGTAGCACCATCTG R: GTCGTATCCAGTGCAGGGTCCGAGGTATTC GCACTGGATACGACTAACCG	60
9	hsa-miR-3165	F: TCGCAGGTGGATGCAATGT R: GTCGTATCCAGTGCAGGGTCCGAGGTATTC GCACTGGATACGACTGAGGT	60
10	hsa-miR-410-3p	F: GCGGCGGAATATAACACAGAT R: GTCGTATCCAGTGCAGGGTCCGAGGTATTC GCACTGGATACGACACAGGC	60
11	hsa-miR-520 h	F: GGCGACAAAGTGCTTCCCT R: GTCGTATCCAGTGCAGGGTCCGAGGTATTC GCACTGGATACGACACTCTA	60
12	hsa-miR-1226-3p	F: GCGTCACCAGCCCTGTGT R: GTCGTATCCAGTGCAGGGTCCGAGGTATTC GCACTGGATACGACCTAGGG	60
13	hsa-miR-373-5p	F: TCAAAATGGGGGCGCTT R: GTCGTATCCAGTGCAGGGTCCGAGGTATTC GCACTGGATACGACGGAAAG	60
14	hsa-miR-1343-3p	F: TCTCCTGGGGCCCGC R: GTCGTATCCAGTGCAGGGTCCGAGGTATTC GCACTGGATACGACGCGAGA	60
15	GAPDH	F: TGCACCACCAACTGCTTAGC R: GGCATGGACTGTGGTCATGAG	60
16	U6	F: CTCGCTTCGGCAGCACA R: AACGCTTCACGAATTTGCGT	60

Note: F, forward primer; R, reverse primer; TM, annealing temperature

### Western blotting

ADO2-iPSCs and NC-iPSCs were homogenized in RIPA buffer (cat. no. KGP702–100; Changchun Keygen Biological Products Co., Ltd.) containing protease inhibitor at 4 °C, and then centrifuged at 16,000 x g for 15 min at 4 °C to extract soluble protein. Protein extracts were quantified using the BCA method and loaded on 12% SDS-gels (20 μg per lane), resolved using SDS-PAGE, and then transferred to nitrocellulose membranes. Following blocking (5% skim milk) for 2 h at room temperature, membranes were incubated overnight at 4 °C with primary antibodies against CLC-7 (1:1000), or α-tubulin (1:2000). Subsequently, the membranes were incubated with HRP-conjugated anti-rabbit secondary antibody (1:1000) at room temperature for 2 h. Signals were visualized using a Quantity One system image analyzer (Bio-Rad Laboratories, Inc.) and densitometry analysis was performed using Image J.

## Results

### Gene variation profiling

**Fig. 1:**
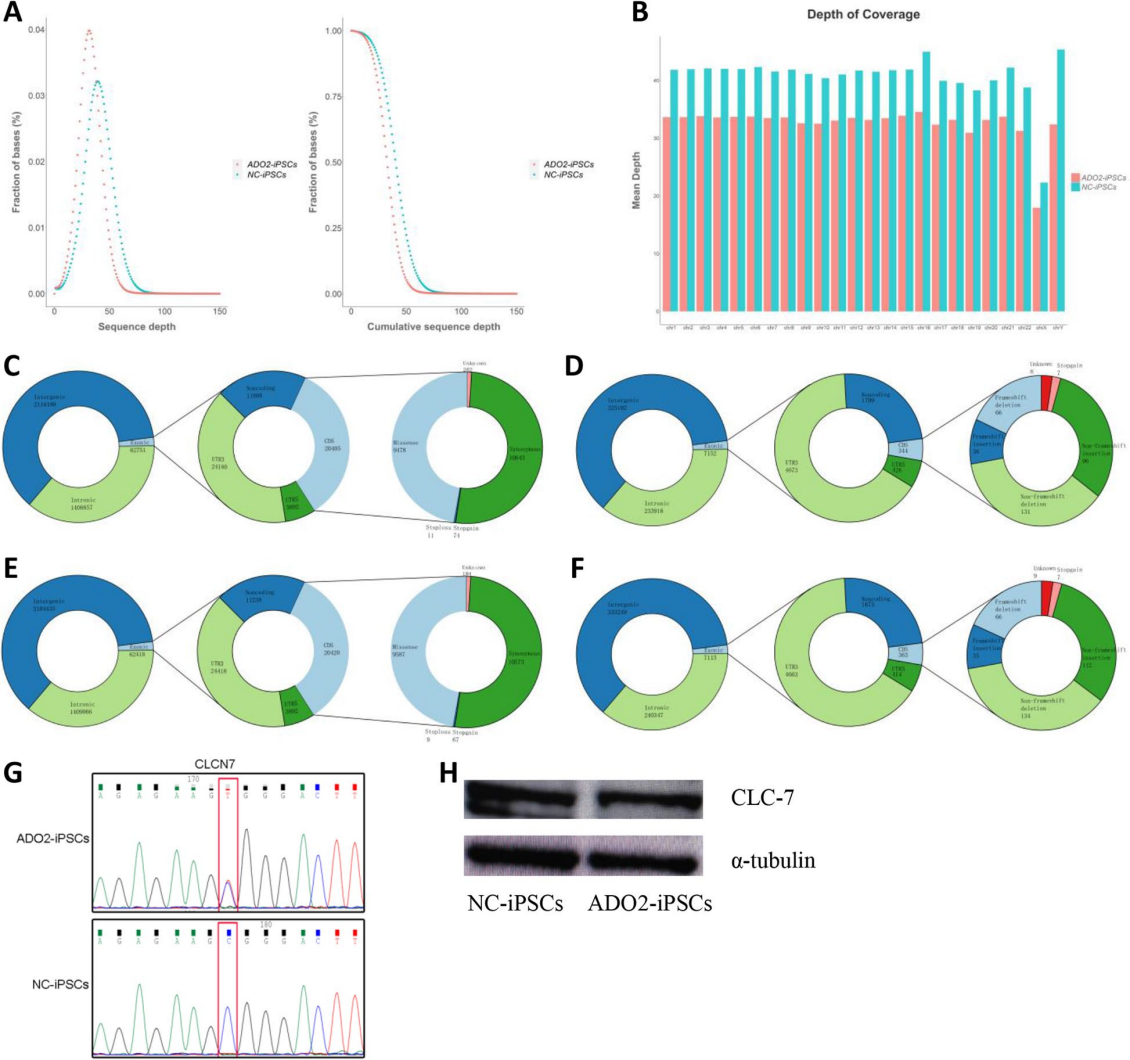
DNA mutation profile of ADO2-iPSCs. (**A**). Statistical analysis of sequence depths for ADO2-iPSCs and NC-iPSCs. (**B**). Mean depth and proportion of covered bases of each chromosome between the two cell lines. (**C**) and (**D**). The detection results of single nucleotide polymorphisms (SNPs) and insertions and deletions (Indels) for ADO2-iPSCs. (**E**) and (**F**). The detection results of single nucleotide polymorphisms (SNPs) and insertions and deletions (Indels) for NC-iPSCs. (**G**). The mutation of CLCN7 (R286W) was confirmed by Sanger sequencing. (**H**). Western blotting analysis of CLC-7 protein expression levels

Genomic DNA was extracted from the two cell lines and sequenced on the Illumina Novaseq™ 6000 platform. A total of 223.83 G raw data was generated, and an average of 373,042,278 raw reads for each cell line (Table S1). The raw reads with adapters, low quality and unknown bases were removed, and we collected clean reads for further statistical analysis. The numbers of clean reads were 664,170,002 and 827,969,924 for ADO2-iPSCs and NC-iPSCs, of which 99.87 and 99.87%, respectively, were accurately aligned to the reference genome (GRCh38) (Table S2). In the ADO2-iPSCs and NC-iPSCs, the sequence depth and coverage of bases are in line with Poisson distribution, and the average depth and coverage of each chromosome of the reference genome (GRCh38) are shown in Fig. 1.

Upon further bioinformatics analysis for clean reads, we identified 3,553,239 and 3,542,578 SNPs for ADO2-iPSCs and NC-iPSCs, and ~ 1.8% (62,715 and 62,418) of these were located in exotic regions. Most SNPs within protein-coding regions were synonymous and missense. Similarly, there were 583,522 and 596,051 InDels for ADO2-iPSCs and NC-iPSCs, and ~ 1.2% (7152 in 583,522, 7113 in 596,051) of these were located in exotic regions (Fig. 1). We next filtered the core osteoporotic genes that were involved in hsa04380 Osteoclast differentiation as candidate genes (Table S3), and they were further confirmed by Sanger sequencing, and Western blotting analysis, such as CLCN7 gene (Fig. 1).

### DNA methylation profiling

**Fig. 2:**
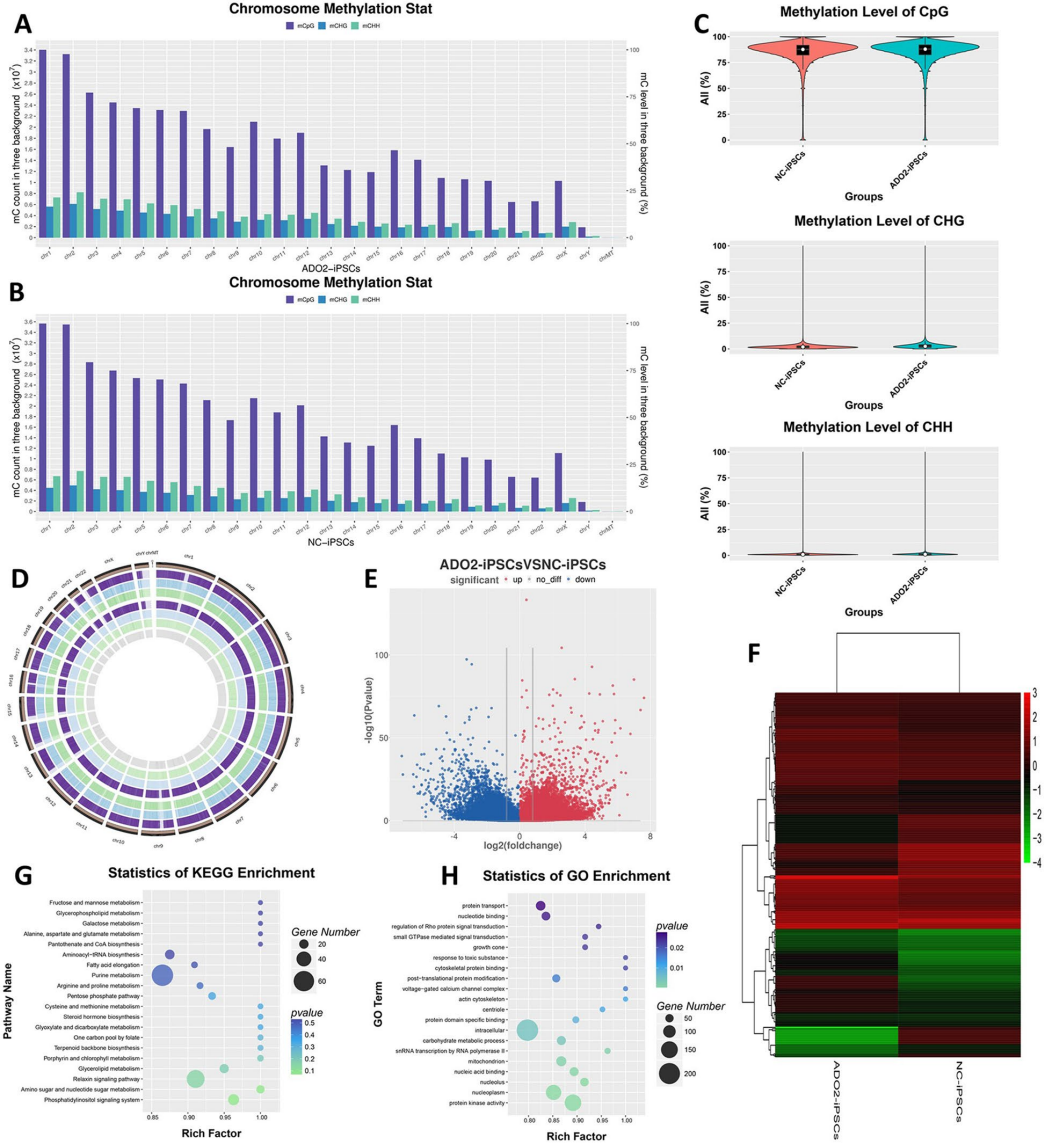
Genome-wide DNA methylation landscape of ADO2-iPSCs. (**A**) and (**B**). The distribution of CpG, CHG and CHH methylation on chromosome for ADO2-iPSCs and NC-iPSCs, respectively. (**C**). The violin plots of CpG, CHG and CHH methylation levels. (**D**). Circos plots of CpG, CHG and CHH methylation levels. (**E**). Volcano plot of differentially expressed methylated genes. (**F**). Clustered heat map of differentially expressed methylated genes between ADO2-iPSCs and NC-iPSCs. (**G**). KEGG pathway enrichment analysis of differentially expressed methylated genes. (**H**). GO enrichment analysis s of differentially expressed methylated genes

To explore the methylation status of ADO2-iPSCs, we sequenced and analysed genome-wide DNA methylation levels of ADO2-iPSCs and NC-iPSCs. In total, 602,933,334 and 601,600,002 raw reads for ADO2-iPSCs and NC-iPSCs were generated, of which 598,992,532 and 598,060,246 valid reads were used for further bioinformatics analysis (Table S4 and S5). The overall DNA methylation levels of mCpG, mCHG and mCHH in the two cell lines were presented in Fig. 2. The figure showed that DNA methylation levels of ADO2-iPSCs were similar to NC-iPSCs. According to the 1000 bp windows, 500 bp overlap and *P* < 0.05, we identified 1,001,943 differentially methylated regions (DMRs). Among these DMRs, 856,823 DMRs were identified as hyper-methylated regions, and 145,120 DMRs were identified as hypo-methylated regions (Fig. 2). Further bioinformatic analysis of the 1,001,943 differentially methylated regions showed that this candidate methylated genes were implicated in various biological functions, and the top 20 GO terms and pathways were presented in Fig. 2. Go enrichment analysis revealed that these differentially expressed methylated genes were highly enriched voltage-gated calcium channel complex and actin cytoskeleton. They are mainly enriched in the following genes: CACNA1S; AC068547.1; CACNA1D; CACNA2D1; CACNA1F; CACNA1B; CACNB2; CACNA2D4; CACNA1G; CACNG4; CACNG1; CACNA1A; CACNG2; LAD1; MPP4; TMEM63B; FILIP1; AC004922.1; ARPC1B; ZNF185; ACTL7B; LSP1; LIMA1; CORO1C; LCP1; DSTN; PDXP.

### m6A methylation profiling

**Fig. 3:**
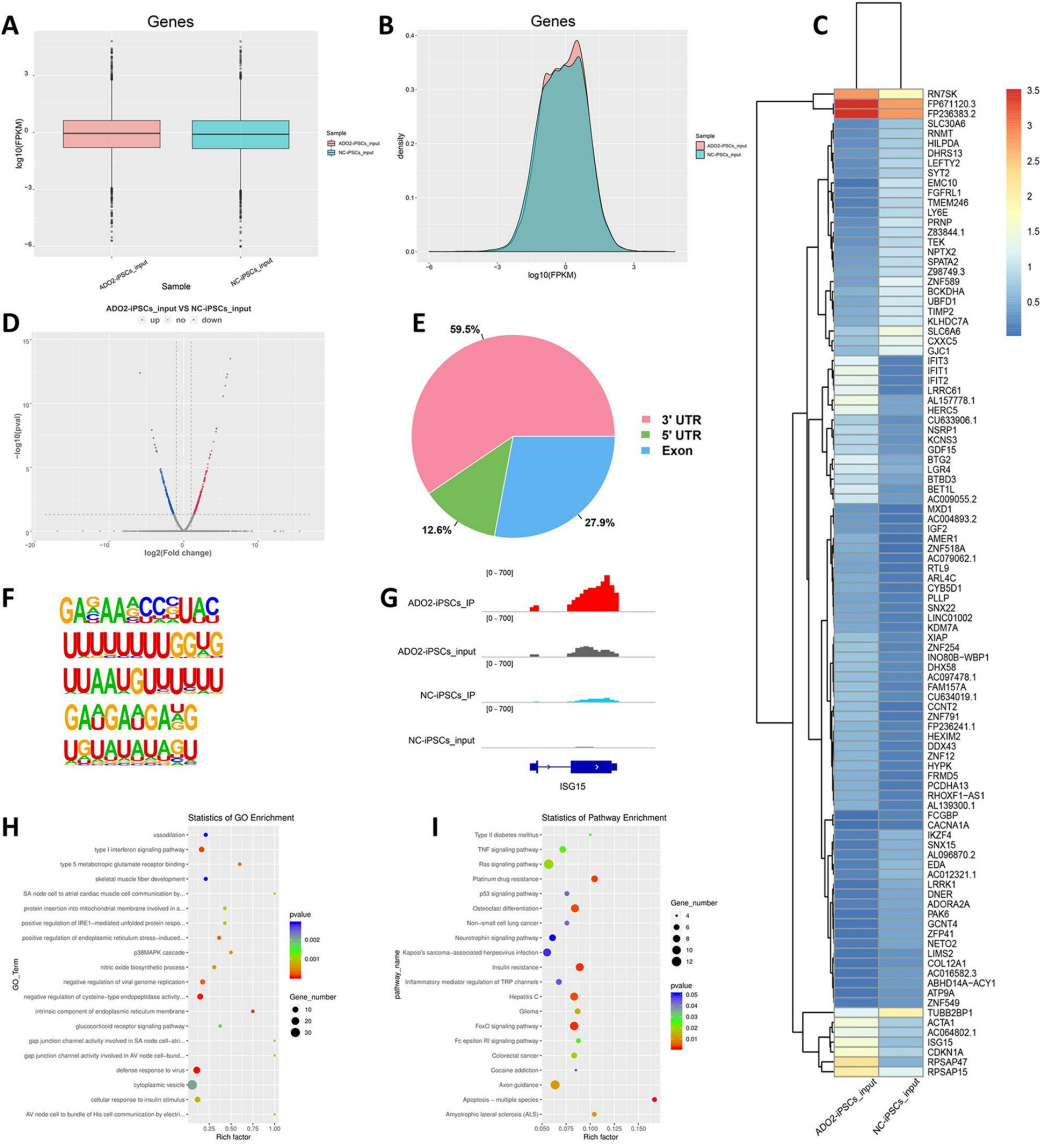
m6A modification signatures of ADO2-iPSCs. (**A**). Boxplots of all genes. (**B**). Density map of all genes. (**C**). Clustered heat map of differentially expressed m6A-methylated genes (DEMGs) between ADO2-iPSCs and NC-iPSCs. (**D**). Volcano plot of the DEMGs. (**E**). The distribution of differential m6A peaks on gene functional elements. (**F**). Sequence logo showing the top motifs enriched across differential m6A peaks identified from ADO2-iPSCs and NC-iPSCs. (**G**). ISG15 m6A level in ADO2-iPSCs sample is higher than NC-iPSCs as shown in Figs. 4 by IGV software. (**H**). GO enrichment analysis of the DEMGs; (**I**). KEGG pathway enrichment analysis of the DEMGs

Like DNA modification, N6-methyladenosine (m6A) is a novel RNA modification that has been extensively studied in plants and animals [28]. To explore the m6A modification levels of ADO2-iPSCs, we sequenced and analysed genome-wide RNA methylation levels of ADO2-iPSCs and NC-iPSCs. In total, the two cell lines were obtained 71,051,686 and 79,256,816 raw data reads, 68,539,884 and 77,263,294 valid reads, and the valid reads accounted for 80.10 and 81.45%, of which the mapping ratio of valid reads in the ADO2-iPSC library and NC-iPSC library were 91.12 and 92.97% in the m6A-seq libraries, respectively. In the RNA-seq library, the two cell lines were obtained 40,503,394 and 46,238,982 raw data reads, 39,422,992 and 45,409,718 valid reads, and the valid reads accounted for 93.84 and 95.23%, of which the mapping ratio of valid reads in the ADO2-iPSC library and NC-iPSC library were 95.28 and 97.09%, respectively (Table S6 and S7). And the regional location of mapped reads is shown in Supplementary Fig. 1. Next, the m6A peaks were further annotated using the ChIPseeker software. And according to *P* < 0.05, we identified only 2984 differential m6A peaks. Among these peaks, 1142 were upregulated and 1842 were downregulated, and the regional location of differential m6A peaks is shown in Fig. 3. Intensive sequence motif analysis for 2984 differential m6A peaks was carried out, and the top five motifs with the smaller *p*-value were presented in the Fig. 3.

And then, the expression abundance of gene was calculated by FPKM. In total, 28,078 m6A-methylated genes were identified, among which 652 m6A-methylated genes were differentially expressed between the two cell lines, and the box plot and of all m6A-methylated gene expression are showed in Figs. 3. According to the |log2(fold change) | > 1 and *p* value< 0.05 criteria, 367 m6A-methylated genes were upregulated and 285 m6A-methylated genes were downregulated, and the volcano plot filtering of the differentially expressed m6A-methylated genes (DEMGs) is presented in Fig. 3. Interestingly, the downregulated genes ISG15 m6A level in ADO2-iPSC sample is higher than NC-iPSC as shown in Figs. 3 by IGV software. Then, we further gathered the 652 DEMGs to conduct GO and KEGG pathway enrichment analyses and found that their biological functions were diverse, and the top 20 significantly GO enrichment terms and enriched pathways were presented in Fig. 3. KEGG pathway enrichment analysis revealed that these DEMGs were highly enriched in the osteoclast differentiation and p53 signalling pathway, which associated with the development of osteopetrosis. In addition, the pathway of osteoclast differentiation is mainly involved in the following candidate genes: CREB1; FOSL1; FOSL2; GAB2; JUNB; MAPK14; PIK3R1; PLCG2; SOCS3. And the FoxO signaling pathway is mainly involved in the following candidate genes: BCL2L11; BCL6; BNIP3; CDKN1A; GADD45B; MAPK14; PDPK1; PIK3R1; RAG1; Z98749.3.

### ceRNA regulatory networks

**Fig. 4:**
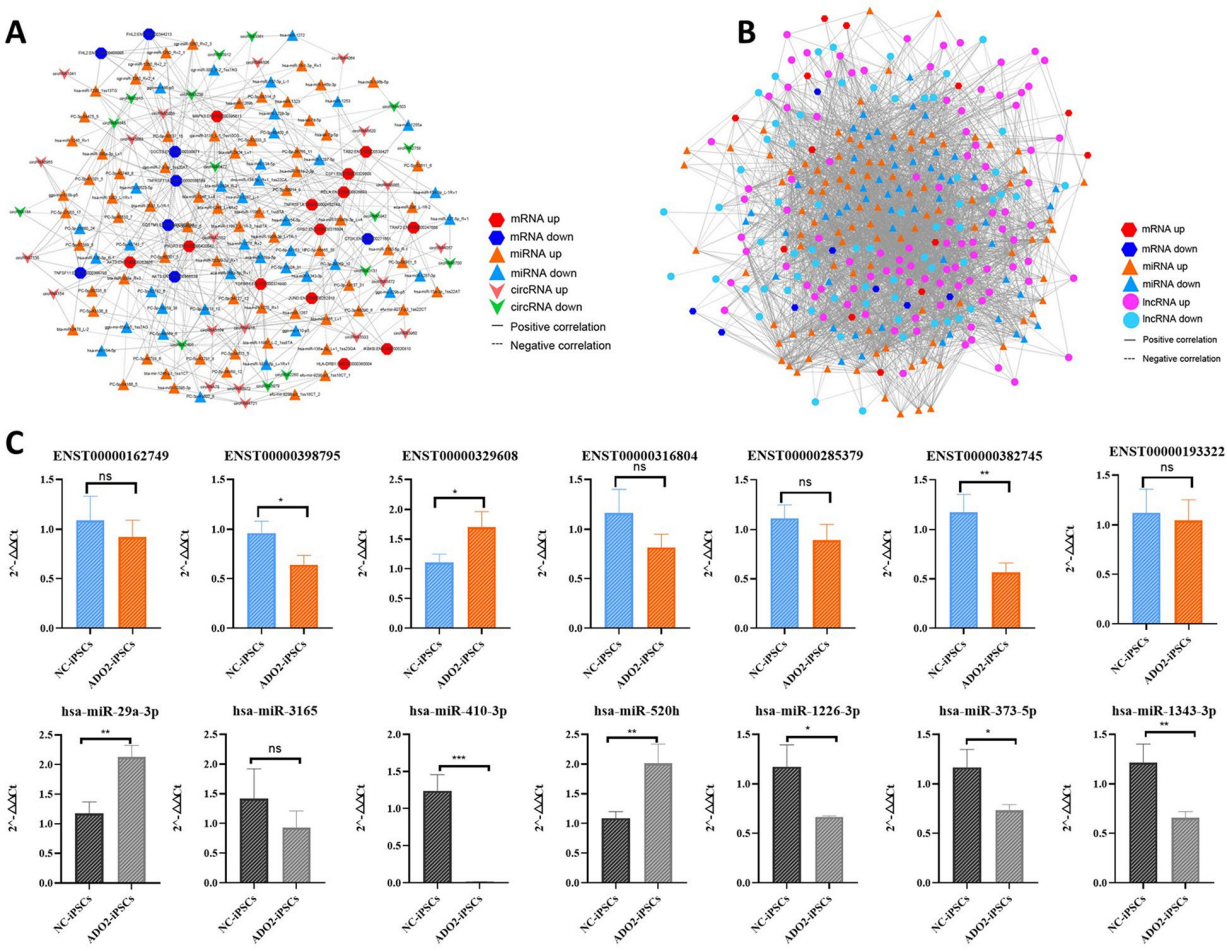
ceRNA networks. (**A**). circRNA-associated-ceRNA networks. (**B**). lncRNA-associated-ceRNA networks. (**C**). Relative expression levels of the sectional genes co-expressed by these ceRNA networks. The data are represented as the means ± SEM of three independent experiments. Asterisks indicate ADO2-iPSCs compared with the controls with *P < 0.05, **P < 0 01, ***P < 0 001 and ****P < 0 0001

To investigate the role of non-coding RNAs in the ADO2-iPSCs, we performed the deep RNA-seq from ADO2-iPSCs and NC-iPSCs to obtain the differential expression profiles of circRNAs, lncRNAs and miRNAs and mRNAs in our previous study. We next used TargetScan (v. 5.0) and miRanda (v. 3.3a) to identify circRNA-miRNA, miRNA-mRNA and lncRNA-miRNA interactions for the construction of ceRNA regulatory network. According to the ceRNAs theory, the relative expressions of miRNAs should be negatively correlated with relative expressions of targeted circRNA/lncRNAs and mRNAs. Therefore, we overlapped the predicted targeting relationships of up-regulated miRNAs with down-regulated circRNA/lncRNAs and mRNAs, and overlapped the predicted targeting relationships of down-regulated miRNAs with up-regulated circRNA/lncRNAs and mRNAs. Finally, we used the Cytoscape (v. 3.5.0) to construct ceRNA regulatory networks that contained two parts: 1). the circRNA-associated-ceRNA networks, which included 15 upregulated circRNAs, 38 downregulated miRNAs and 13 upregulated mRNAs, as well as 20 downregulated circRNAs, 69 upregulated miRNAs and 8 downregulated mRNAs; 2). the lncRNA-associated-ceRNA networks, which included 97 upregulated lncRNAs, 53 downregulated miRNAs and 13 upregulated mRNAs, as well as 63 downregulated lncRNAs, 96 upregulated miRNAs and 8 downregulated mRNAs (Fig. 4). In addition, the sectional genes co-expressed by these ceRNA networks were further confirmed by qRT-PCR, and the results showed that the relative expression of genes was basically consistent with the results of high-throughput sequencing (Fig. 4).

### Proteomic profiling

**Fig. 5:**
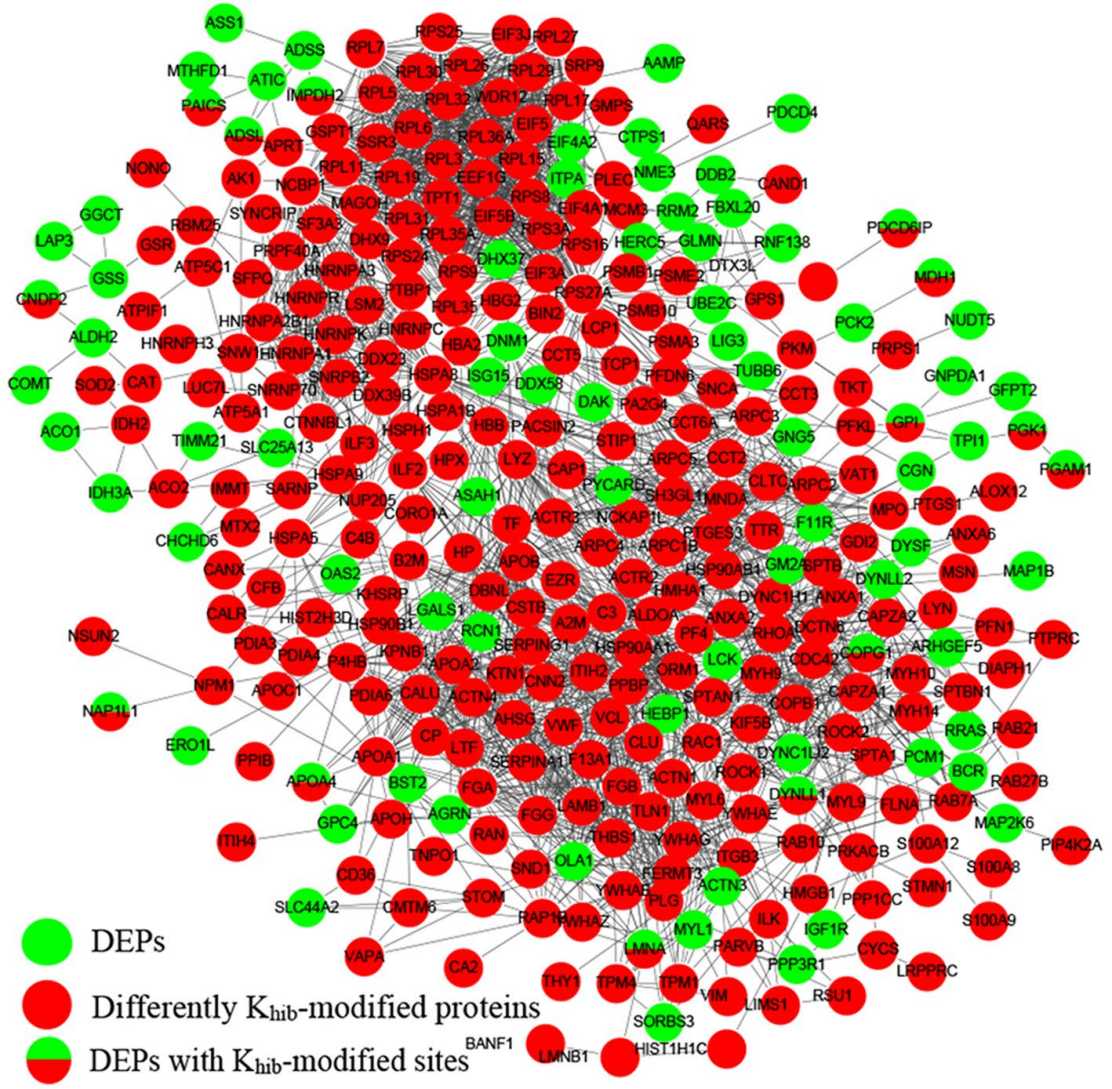
The potential regulatory network among the candidate proteins, DEPs and DEKMPs

To understand the molecular characterization of ADO2, we performed an in-depth proteomic profiling from the two cell lines to obtain the differential expression profiles of proteins and K_hib_-modified proteins in our previous research [1]. Further advanced intersection analysis for 177 differentially expressed proteins (DEPs) and 410 differentially expressed K_hib_-modified proteins (DEKMPs), we collected 15 DEPs with differentially K_hib_-modified sites as candidate proteins for additional analysis (Table 2). We next gathered the 15 candidate proteins to perform GO and KEGG pathway enrichment analysis. The majority of GO terms including the candidate proteins are presented in Fig. S2. In the cellular component, the candidate proteins were highly enriched in cytosol (GO:0005829). In the biological process, the terms of nucleoside phosphate metabolic process (GO:0006753), ribose phosphate metabolic process (GO:0019693) and oxidoreduction coenzyme metabolic process (GO:0006733) were the most enriched. For the KEGG pathway analysis, the only one significantly enriched pathway (hsa00010 Glycolysis / Gluconeogenesis) were identified for these candidate proteins (Supplementary Fig. 2). The regulation network among the candidate proteins, DEPs and DEKMPs were constructed as a whole, and the interaction between them may affect the pathogenesis of osteopetrosis (Fig. 5).

**Table 2:** Summary of the candidate proteins from proteomic profile

Index	Protein accession	Protein description	Fold change	Up/ down	P value	Gene name
1	P22234	Multifunctional protein ADE2 OS=*Homo sapiens* OX = 9606 GN=PAICS	0.8	Down	0.000035892	PAICS
2	P06727	Apolipoprotein A-IV OS=*Homo sapiens* OX = 9606 GN = APOA4	0.7	Down	0.035622	APOA4
3	P12268	Inosine-5′-monophosphate dehydrogenase 2 OS=*Homo sapiens* OX = 9606 GN=IMPDH2	0.814	Down	0.00060007	IMPDH2
4	P40925	“Malate dehydrogenase, cytoplasmic OS=*Homo sapiens* OX = 9606 GN = MDH1”	0.794	Down	1.2969E-09	MDH1
5	P30566	Adenylosuccinate lyase OS=*Homo sapiens* OX = 9606 GN = ADSL	0.692	Down	1.7211E-09	ADSL
6	P55209	Nucleosome assembly protein 1-like 1 OS=*Homo sapiens* OX = 9606 GN=NAP1L1	0.796	Down	0.027664	NAP1L1
7	Q8WUM4	Programmed cell death 6-interacting protein OS=*Homo sapiens* OX = 9606 GN=PDCD6IP	0.764	Down	0.00058313	PDCD6IP
8	Q96KP4	Cytosolic non-specific dipeptidase OS=*Homo sapiens* OX = 9606 GN=CNDP2	0.792	Down	1.0982E-06	CNDP2
9	P06744	Glucose-6-phosphate isomerase OS=*Homo sapiens* OX = 9606 GN = GPI	0.81	Down	3.2818E-08	GPI
10	P00558	Phosphoglycerate kinase 1 OS=*Homo sapiens* OX = 9606 GN=PGK1	0.798	Down	2.48268E-12	PGK1
11	Q9Y678	Coatomer subunit gamma-1 OS=*Homo sapiens* OX = 9606 GN=COPG1	1.523	Up	0.00020409	COPG1
12	P02545	Prelamin-A/C OS=*Homo sapiens* OX = 9606 GN = LMNA	1.3	Up	6.8335E-10	LMNA
13	P00338	L-lactate dehydrogenase A chain OS=*Homo sapiens* OX = 9606 GN = LDHA	0.73	Down	2.13718E-13	LDHA
14	P18669	Phosphoglycerate mutase 1 OS=*Homo sapiens* OX = 9606 GN=PGAM1	0.772	Down	3.5611E-06	PGAM1
15	P63167	“Dynein light chain 1, cytoplasmic OS=*Homo sapiens* OX = 9606 GN=DYNLL1”	0.813	Down	0.0032784	DYNLL1

### Integration analysis of multi-omics datasets

**Fig. 6:**
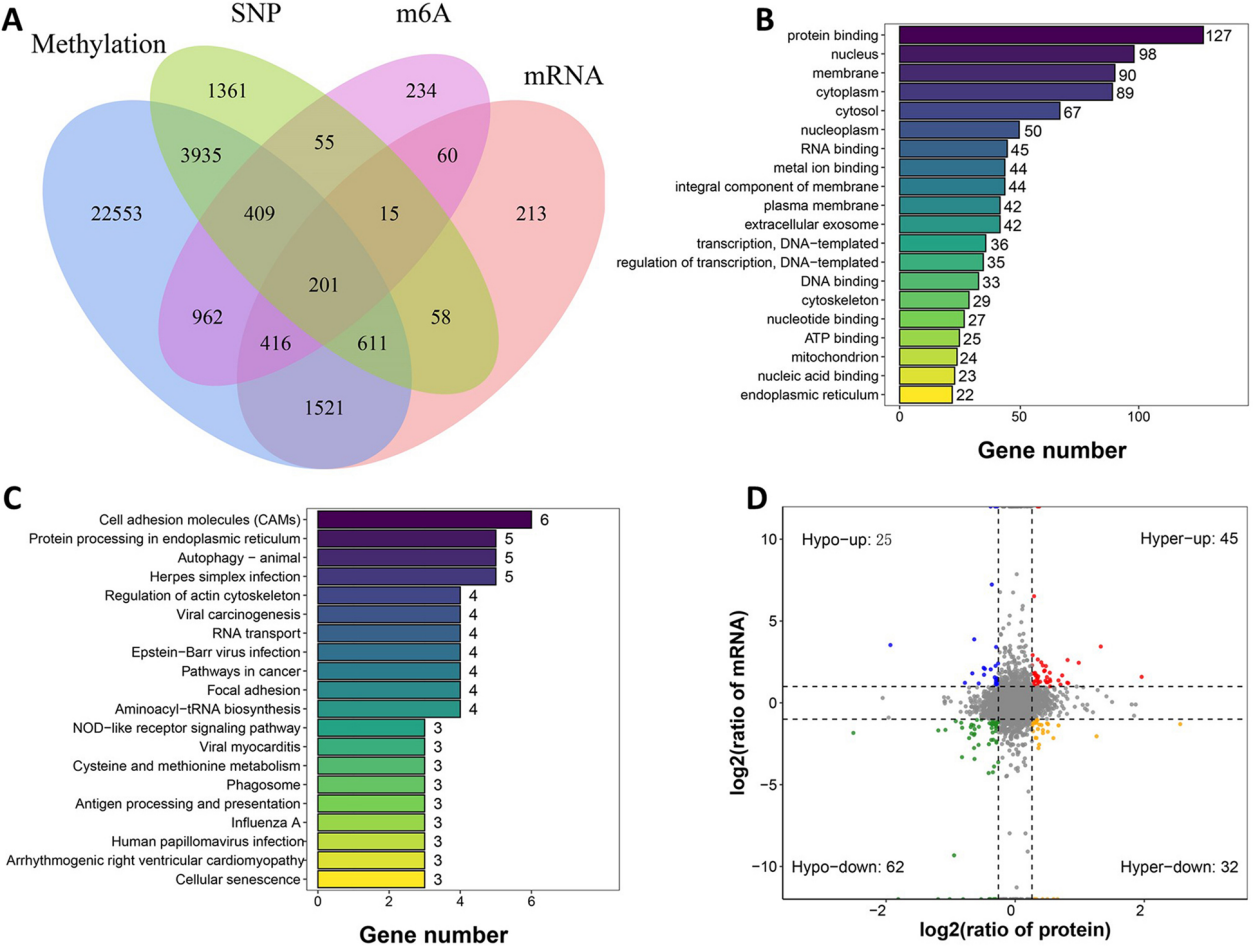
Integration analysis of multi-omics datasets. (**A**) Venn diagram of the combined analysis of four omics, including whole genome re-sequencing, DNA methylation, whole transcriptome sequencing and N6-methyladenosine. (**B**). GO enrichment analysis of the overlapped genes with four omics. (**C**). KEGG pathway enrichment analysis of the overlapped genes with four omics. (**D**). Spearman correlation analysis between quantitative data of mRNA and protein fold changes (*n* = 164 mRNA-protein pairs)

Some evidence indicated that the methylation changes were negatively correlated with gene expression [8]. And thus, we overlapped the up-regulated DMRs with down-regulated mRNAs and proteins, and overlapped the down-regulated DMRs with up-regulated mRNAs and proteins. Indeed, we found that up-regulated DMRs with were significantly associated with anticorrelated gene expression in the ADO2-iPSCs (Supplementary Fig. 3). In addition, pairwise comparisons indicated a global positive correlation between transcription and proteome (Fig. 6). Further intersection analysis for four omics, including whole genome re-sequencing, DNA methylation, whole transcriptome sequencing and N6-methyladenosine, we collected 201 overlapped genes for bioinformatics analysis. Gene ontology (GO) enrichment analysis indicated that the overlapped genes were significantly enriched in protein binding, nucleus, membrane, cytoplasm and cytosol. In addition, KEGG pathway enrichment analysis revealed that the overlapped genes were mainly enriched in cell adhesion molecules (CAMs), Protein processing in endoplasmic reticulum, and Autophagy-animal (Fig. 6). Interestingly, the pathway of cell adhesion molecules is mainly involved in the following overlapped genes: SDC3, ITGA6, HLA-A, CD276, CDH3, and PVR. The pathway of protein processing in endoplasmic reticulum is mainly involved in the following overlapped genes: SSR1, CAPN1, HSP90AA1, EIF2AK4, NSFL1C. And the pathway of Focal adhesion is mainly involved in the following overlapped genes: ITGA6, FLNB, MYLK, FLNA.

## Discussion

Autosomal dominant osteopetrosis type 2 (ADO2) is one of the rarest human inherited bone diseases that is characterized by osteoclast abnormalities, and the clinical manifestations of ADO2 extend beyond the skeleton, affecting several other organs, such as brain, lungs, kidneys and muscles [2, 37]. The pathogenesis of ADO2 remains elusive due to the lack of clinical samples and mouse models, and there is no specialized treatment for human CLCN7-dependent ADO2 [16]. Recently, the autosomal dominant osteopetrosis type 2 disease-specific induced pluripotent stem cells (ADO2-iPSCs) were generated, which carried the same genetic background with the ADO2 patients and may be a useful tool for ADO2 studies [1]. Some evidence has strongly indicated that gene expression changes affecting cellular processes in human diseases are also present in these undifferentiated disease-specific iPSCs [38–41]. For example, Chae et al. performed quantitative proteomic analysis of HD-iPSC derived from a human Huntington’s (HD) disease patients, and they found that the undifferentiated HD-iPSCs provided valuable information to understand the pathogenesis of HD [38]. Szlachcic and colleagues performed another study on the naïve mouse HD YAC128 iPSCs and two types of human HD iPSC generated from HD and juvenile-HD patients, they found that a number of changes affecting cellular processes in HD were also present in undifferentiated HD-iPSCs [39]. In addition, as far as we know, this is the first time that we have used an osteopetrotic iPSC model (ADO2-iPSCs) to explore the pathogenesis of osteopetrosis [1], and found that protein expression changes affecting osteoclast function were also present in undifferentiated ADO2-iPSCs, and the pathogenesis of ADO2 may begin early in life. It may be a very interesting point for our future study using the generated ADO2-iPSCs. However, there are the following limitations for the samples in this study: firstly, we have limited number of patients with ADO2 in the orthopedics department of Shenzhen People’s Hospital [1]; secondly, we have very restricted criteria for volunteer recruitment, and all participants were clinically identified and diagnosed according to the standard spine and pelvis radiographs and genotyping. Therefore, this was a reason that the sample size of ADO2-iPSCs group is smaller.

It is noteworthy that the susceptibility in human genetic disease is commonly associated with DNA mutations, such as single nucleotide polymorphisms (SNPs) and insertion and deletions (InDels). In this study, we detected the vast majority of DNA mutations from the ADO2-iPSCs, including SNPs and Indels, which may affect protein structure and stability. Some models were developed for determining the mechanism of each DNA mutation at the protein level base on the vitro mutagenesis studies and the protein structural context of each mutation [42]. Approximately 90 % of the known disease-causing missense mutations affected protein stability through a variety of energy related factors, which may be playing important roles in the pathologic processes of diseases [42]. ADO2 is a typical human genetic disease, and dozens of studies have indicated that the mutation CLCN7 (R286W) is a disease-causing heterozygous mutation in many osteopetrosis families [15, 43], and confirmed by Sanger Sequencing in our present study. From the literature of the mutation CLCN7 (R286W), we can learn that the mutation CLCN7 (R286W) may lead to the defects in translations of ClC-7, and the affected amino acid is R286. However, there was no difference in the expression of ClC-7 between the two cell lines in the proteomic profile, so we speculated that the heterozygous mutation of CLCN7 (R286) might not affect its expression and might change its protein structure. Although almost all of the published studies are based on the data of DNA sequencing and bioinformatics analysis, it may be reasonable to believe that defect in translation is one of the most important cause of osteopetrosis. Furthermore, the proteomic profile indicated that the K_hib_-modified proteins has a significant effect on osteoclast function, such as the P00918 (carbonic anhydrase 2, CA2) with four differently K_hib_-modified sites [1], and the cross-talk between the DEPs and differentially expressed K_hib_-modified proteins may plays an important role in the pathogenesis of osteopetrosis.

The changes in DNA methylation profiles suggest epigenetic alteration, which associated with disease susceptibility [44–46], and may be a promising biomarker for clinical applications [47, 48]. In this study, we found that the DNA methylation levels in the two cell lines were similar, with CpG methylation levels higher than CHG and CHH methylation levels. Like DNA modification, N6-methyladenosine (m6A) is a novel RNA modification that has been extensively studied in plants and animals [28]. We next obtain the m6A methylation profiles from the two cell lines using MeRIP-seq and RNA-seq method. In this study, 2984 differential m6A peaks were identified, and they were mainly located in 3’UTRs, which is consistent with previous reports [28]. Noteworthy, we observed that the m6A modification levels of ISG15 in ADO2-iPSCs is higher than NC-iPSCs in the present study, and its protein expression was also high in the proteomic profile [1], which is involved in regulating the RIG-I-like receptor signalling pathway and bone formation [49]. Further functional analysis of the KEGG pathway indicated that these m6A-modified genes were involved in the Osteoclast differentiation and p53 signalling pathway, which may regulate osteoclast development and differentiation [50, 51]. Furthermore, noncoding RNAs can regulate osteoclast differentiation and function by changing the microenvironment of osteoclastogenesis [52, 53].

## Conclusions

In summary, the global multi-omics signatures for ADO2-iPSCs and NC-iPSCs generated in this study represent not only a comprehensive data resource but also provide additional biological insights underlying clinical features of osteopetrosis. In addition, our results indicate that the pathogenesis of ADO2 may begin early in life.

## Supplementary Information

**Additional file 1.**

## Data Availability

The raw and processed data from small RNA sequencing and ribosomal RNA-depleted sequencing in this study has been deposited with the Gene Expression Omnibus under accession number GSE133937 and GSE140132 respectively. And the raw data from Proteomic profiling has been deposited to the ProteomeXchange Consortium via the PRIDE partner repository with the dataset identifier PXD014227.

## Abbreviations

ADO2: Autosomal dominant osteopetrosis type II
ADO2-iPSCs: autosomal dominant osteopetrosis type II iPSCs
NC-iPSCs: normal control iPSCs
m6A: N6-methyladenosine
SNPs: single nucleotide polymorphisms
InDels: insertion and deletions
DMRs: Differentially methylated regions
DEMGs: differentially expressed m6A-modified genes
ARO: autosomal recessive osteopetrosis
GO: gene ontology
KEGG: kyoto encyclopedia of genes and genomes
qRT-PCR: Quantitative real-time polymerase chain reaction
ncRNAs: noncoding RNAs
ceRNA: competing endogenous RNA
DEPs: differentially expressed proteins
DEKMPs: differentially expressed K_hib_-modified proteins
HD: human Huntington’s disease
CA2: carbonic anhydrase 2
