# Supplementary material for: Multiomics landscape of the autosomal dominant osteopetrosis type II disease-specific induced pluripotent stem cells
Source: Hereditas. 2021 Oct 27;158:40. doi: 10.1186/s41065-021-00204-x (PMC8549315; doi:10.1186/s41065-021-00204-x)
Supplement: Supplementary file 1 — Additional file 1. [file 41065_2021_204_MOESM1_ESM.docx]

**Supplementary Material**

**Article Title:** Multiomics landscape of the autosomal dominant osteopetrosis type II disease-specific induced pluripotent stem cells

**Author Data:** Chunhong Li^1^, Yu Shangguan ^2, 3^, Peng Zhu^2^, Weier Dai^4^, Donge Tang^#, 2^, Minglin Ou^#, 1^, Yong Dai^#, 2^

^1^ Central Laboratory, Guangxi Health Commission Key Laboratory of Glucose and Lipid Metabolism Disorders, The Second Affiliated Hospital of Guilin Medical University, Guilin, Guangxi, P. R. 541199, China;

^2^ Clinical Medical Research Center, Guangdong Provincial Engineering Research Center of Autoimmune Disease Precision Medicine, Shenzhen Engineering Research Center of Autoimmune Disease, The Second Clinical Medical College of Jinan University, The First Affiliated Hospital of Southern University of Science and Technology, Shenzhen People’s Hospital, Shenzhen, Guangdong 518020, P.R. China;

^3^ Guangxi Key Laboratory of Metabolic Disease Research, Central Laboratory of Guilin NO. 924 Hospital, Guilin, 541002, P.R. China;

^4^ College of Natural Science, University of Texas at Austin, Austin, Texas, 78712, United States of America.

**^#^ Corresponding authors**: Yong Dai, MD, PhD, Doctor, Clinical Medical Research Center, Guangdong Provincial Engineering Research Center of Autoimmune Disease Precision Medicine, Shenzhen Engineering Research Center of Autoimmune Disease, The Second Clinical Medical College of Jinan University, The First Affiliated Hospital of Southern University of Science and Technology, Shenzhen People’s Hospital, Shenzhen, Guangdong 518020, P.R. China.

E-mail: daiyong22@aliyun.com

Telephone: +86-0755-25533000-2780

Fax: +86 755-25626750

Minglin Ou, PhD, Central Laboratory, Guangxi Health Commission Key Laboratory of Glucose and Lipid Metabolism Disorders, The Second Affiliated Hospital of Guilin Medical University, Guilin, Guangxi, P. R. 541199, China.

E-mail: minglinou@163.com Telephone: +86-0773-5593599

Fax: +86-0773-5593599

Donge Tang, PhD, Clinical Medical Research Center, Guangdong Provincial Engineering Research Center of Autoimmune Disease Precision Medicine, Shenzhen Engineering Research Center of Autoimmune Disease, The Second Clinical Medical College of Jinan University, The First Affiliated Hospital of Southern University of Science and Technology, Shenzhen People’s Hospital, Shenzhen, Guangdong 518020, P.R. China.

E-mail: donge66@126.com

Telephone: +86-0755-25533000-2780

Fax: +86 755-25626750

**Email addresses**: Chunhong Li: 842567961@qq.com;

Yu Shangguan: 1026766505@qq.com;

Peng Zhu: 546850405@qq.com;

Weier Dai: Will300530@gmail.com;

Donge Tang: donge66@126.com;

Minglin Ou: minglinou@163.com;

Yong Dai: daiyong22@aliyun.com.


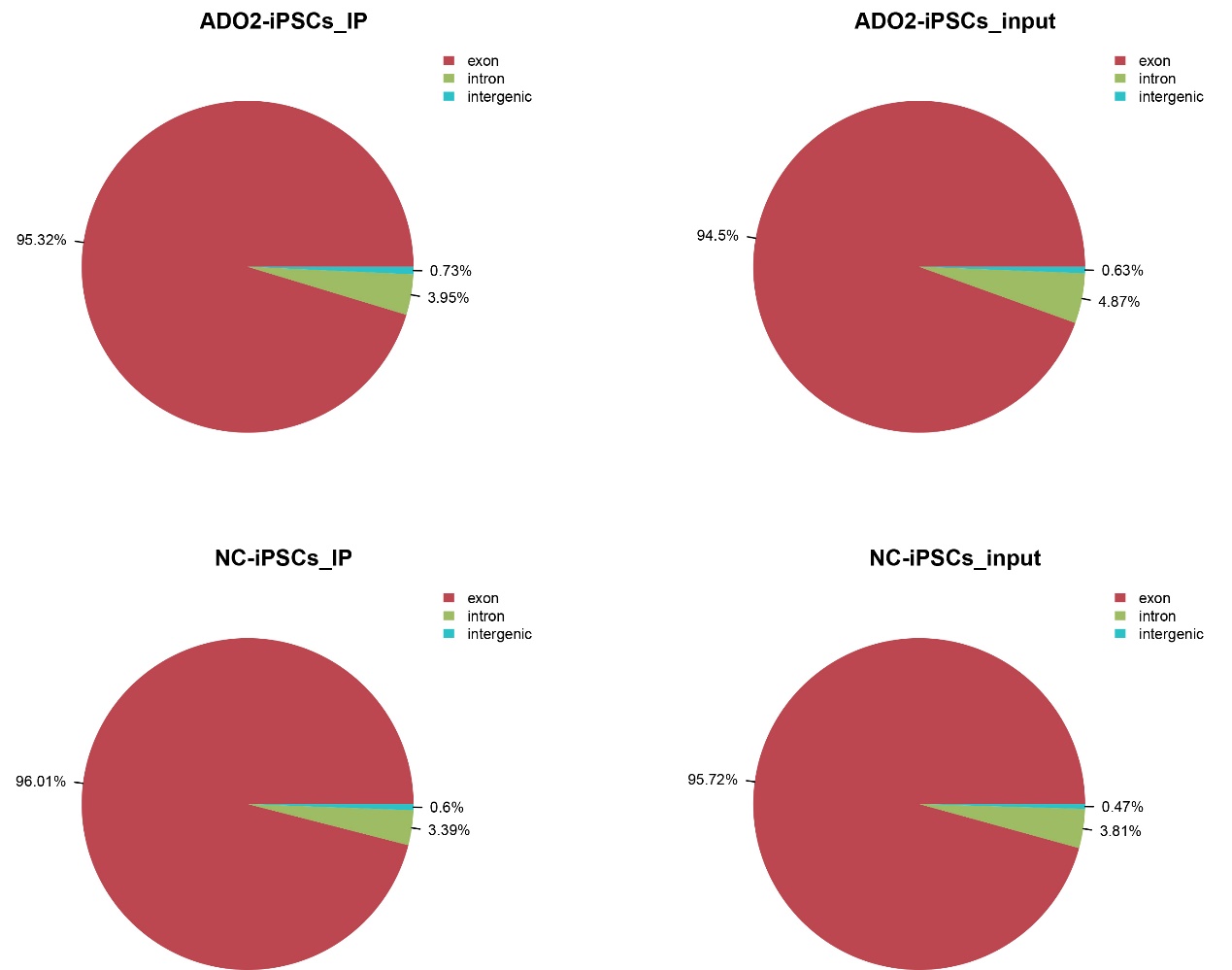


**Supplementary Figure 1**. Refer to the genome to compare the regional distribution.


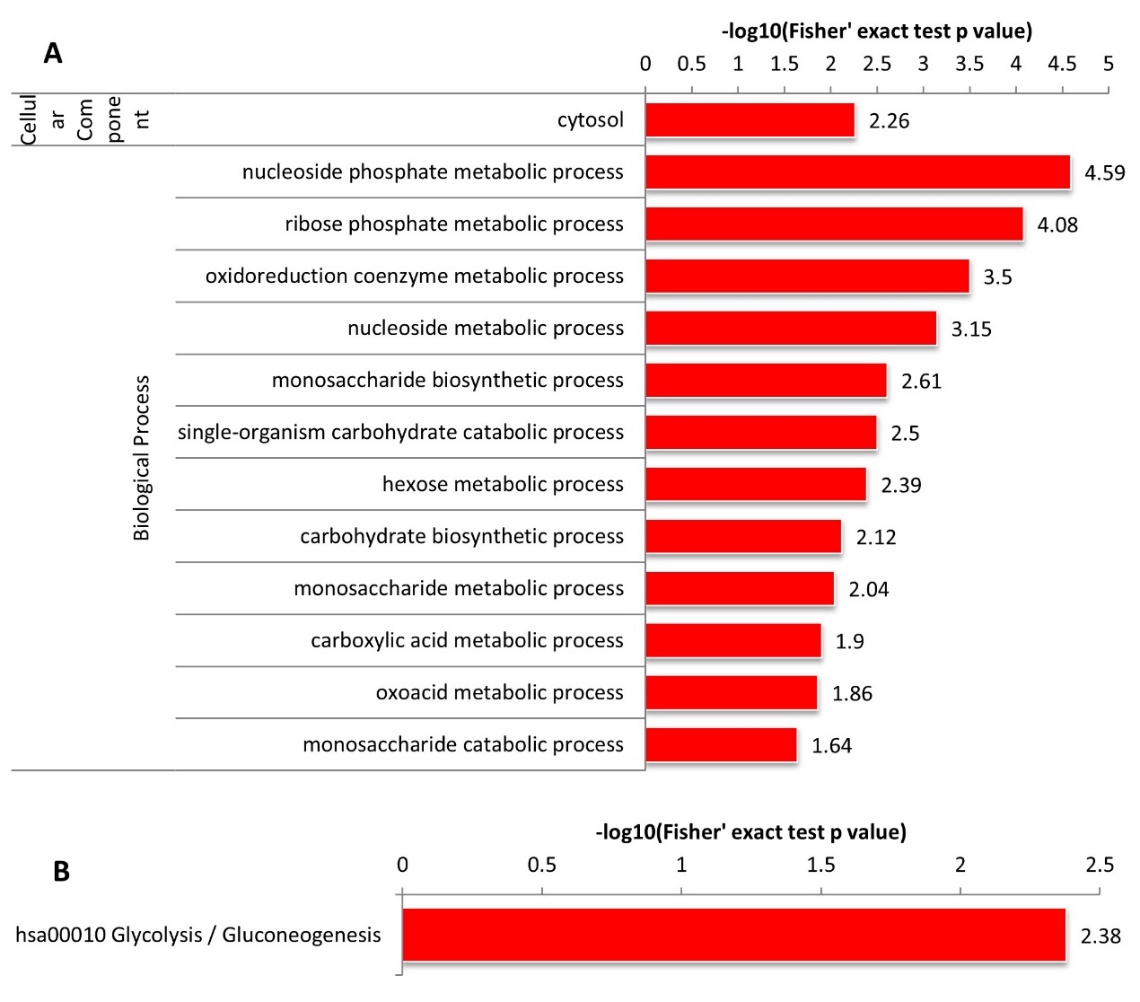
 **Supplementary Figure 2**. Functional enrichment analysis of the differentially expressed proteins with differentially Khib-modified sites in the ADO2-iPSCs. (A). GO-based functional enrichment analysis. (B). KEGG-based functional enrichment analysis.


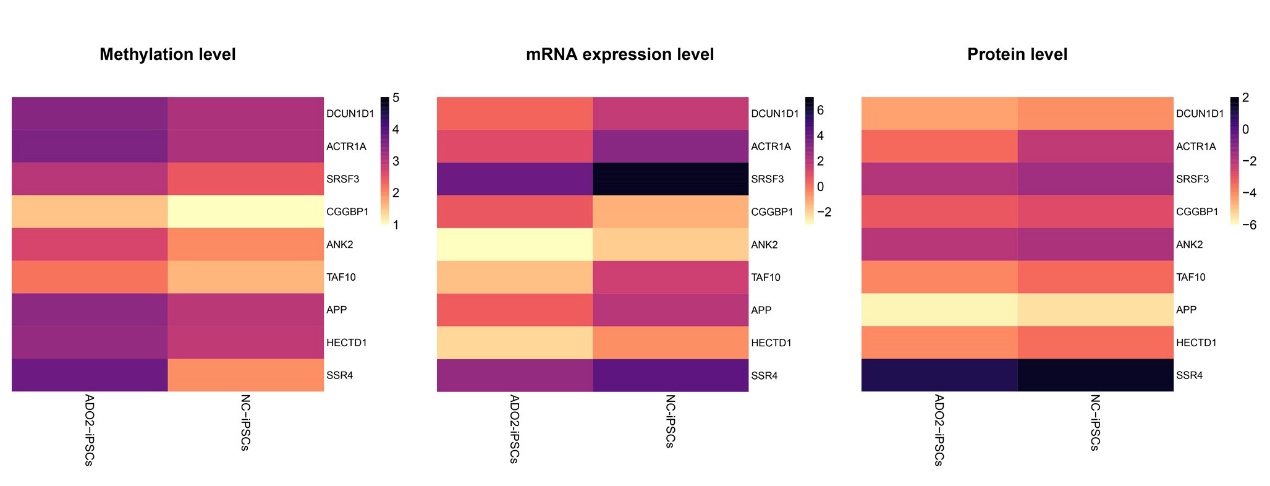


**Supplementary Figure 3**. Clustered heat map of differentially expressed methylated genes, mRNAs and proteins between ADO2-iPSCs and NC-iPSCs.

**Table S1. Statistical analysis of whole genome re-sequencing data**

| Sample name | Raw reads | Raw data(G) | Effective (%) | Error (%) | Q20 (%) | Q30 (%) | GC (%) |
| --- | --- | --- | --- | --- | --- | --- | --- |
| NC-iPSCs | 413993008 | 124.2 | 100 | 0.03 | 97.85 | 93.88 | 40.12 |
| ADO2-iPSCs | 332091548 | 99.63 | 100 | 0.03 | 97.94 | 94.1 | 40.21 |

**Table S2. Alignment data summary**

| Sample | NC-iPSCs | ADO2-iPSCs |
| --- | --- | --- |
| Total | 827,969,924 (100%) | 664,170,002 (100%) |
| Duplicate | 118,502,468 (14.31%) | 84,716,568 (12.76%) |
| Mapped | 826,910,638 (99.87%) | 663,330,652 (99.87%) |
| Properly mapped | 814,791,656 (98.41%) | 652,976,228 (98.31%) |
| PE mapped | 826,299,252 (99.80%) | 662,850,374 (99.80%) |
| SE mapped | 1,222,772 (0.15%) | 960,556 (0.14%) |
| With mate mapped to a different chromosome | 8,926,156 (1.08%) | 7,344,416 (1.11%) |
| With mate mapped to a different chromosome ((map Q>=5)) | 7,345,120 (0.89%) | 5,960,859 (0.90%) |
| Average sequencing depth | 40.59 | 32.56 |
| Coverage | 100% | 100% |
| Coverage at least 4X | 99% | 99% |
| Coverage at least 10X | 99% | 98% |
| Coverage at least 20X | 94% | 89% |

**Table S3. Summary of candidate single nucleotide polymorphisms**

| CHROM | POS | REF | ALT | QUAL | Gene Name |
| --- | --- | --- | --- | --- | --- |
| chr1 | 109924087 | T | C | 511.77 | CSF1 |
| chr1 | 198536700 | C | T | 348.77 | ATP6V1G3 |
| chr16 | 1456173 | G | A | 253.77 | CLCN7 |
| chr18 | 62360008 | C | T | 556.77 | TNFRSF11A |
| chr19 | 54219161 | A | C | 1141.77 | LILRB3 |
| chr19 | 54222756 | T | C | 183.77 | LILRB3 |
| chr19 | 54240923 | G | C | 154.77 | LILRA6 |
| chr19 | 54242117 | T | C | 302.77 | LILRA6 |
| chr19 | 54242175 | C | A | 339.77 | LILRA6 |
| chr2 | 10768767 | A | G | 495.77 | ATP6V1C2 |
| chr2 | 70935956 | T | C | 460.77 | ATP6V1B1 |
| chr7 | 138733046 | A | G | 432.77 | ATP6V0A4 |
| chr7 | 138771243 | A | G | 424.77 | ATP6V0A4 |

**Table S4. Statistical analysis of whole-genome bisulphite sequencing data**

| Sample name | Raw reads | Raw Base (G) | Valid reads | Valid Base (G) | Depth (x) | Valid (%) | Q20 (%) | Q30 (%) |
| --- | --- | --- | --- | --- | --- | --- | --- | --- |
| NC-iPSCs | 601600002 | 90.24 | 598060246 | 83.54 | 99.41 | 98.88 | 97.27 | 23.64 |
| ADO2-iPSCs | 602933334 | 90.44 | 598992532 | 85.95 | 99.35 | 98.81 | 97.14 | 22.45 |

**Table S5. Statistical analysis of whole-genome bisulphite sequencing mapping data**

| Sample | NC-iPSCs | ADO2-iPSCs |
| --- | --- | --- |
| Total read pairs | 598992532 | 598060246 |
| Unique mapped reads | 454842611 | 442947255 |
| Unique reads mapping rate (%) | 75.93 | 74.06 |
| Duplication reads | 69376095 | 68966129 |
| Duplication rate (%) | 11.58 | 11.53 |
| mean of C coverage (%) | 31.47 | 31.26 |
| >=2xC coverage (%) | 25.66 | 24.82 |
| >=5xC coverage (%) | 14.49 | 12.45 |
| >=10xC coverage (%) | 6.16 | 4.63 |
| >=15xC coverage (%) | 2.64 | 1.92 |

**Table S6. Summary of reads quality control from IP and input RNA-seq**

| Sample | Raw Reads | Raw Bases | Valid Reads | Valid Bases | Valid (%) | Q20 (%) | Q30 (%) | GC (%) |
| --- | --- | --- | --- | --- | --- | --- | --- | --- |
| ADO2-iPSCs_input | 40503394 | 6.08G | 39422992 | 5.70G | 93.84 | 98.54 | 95.65 | 49.56 |
| ADO2 -iPSCs _IP | 71051686 | 10.73G | 68539884 | 8.59G | 80.10 | 98.22 | 95.32 | 51.73 |
| NC- iPSCs _input | 46238982 | 6.94G | 45409718 | 6.61G | 95.23 | 98.76 | 96.05 | 49.91 |
| NC- iPSCs _IP | 79256816 | 11.97G | 77263294 | 9.75G | 81.45 | 98.33 | 95.52 | 52.98 |

**Table S7. Summary of reads mapping to the human reference genome**

| Sample | ADO2-iPSCs_IP | ADO2-iPSCs_input | NC-iPSCs_IP | NC-iPSCs_input |
| --- | --- | --- | --- | --- |
| Valid reads | 68539884 | 39422992 | 77263294 | 45409718 |
| Mapped reads | 62450926 (91.12%) | 37561719 (95.28%) | 71831114 (92.97%) | 44087638 (97.09%) |
| Unique Mapped reads | 53054190 (77.41%) | 27721583 (70.32%) | 61949282 (80.18%) | 33464535 (73.69%) |
| Multi Mapped reads | 9396736 (13.71%) | 9840136 (24.96%) | 9881832 (12.79%) | 10623103 (23.39%) |
| PE Mapped reads | 60009682 (87.55%) | 37129224 (94.18%) | 69055438 (89.38%) | 43613046 (96.04%) |
| Reads map to sense strand | 29159349 (42.54%) | 17310687 (43.91%) | 33880050 (43.85%) | 20744847 (45.68%) |
| Reads map to antisense strand | 29266625 (42.70%) | 17331057 (43.96%) | 33995518 (44.00%) | 20773487 (45.75%) |
| Non-splice reads | 43894263 (64.04%) | 22041944 (55.91%) | 50715444 (65.64%) | 26059320 (57.39%) |
